# Botulinum Neurotoxin Chimeras Suppress Stimulation by Capsaicin of Rat Trigeminal Sensory Neurons In Vivo and In Vitro

**DOI:** 10.3390/toxins14020116

**Published:** 2022-02-04

**Authors:** Caren Antoniazzi, Mariia Belinskaia, Tomas Zurawski, Seshu Kumar Kaza, J. Oliver Dolly, Gary W. Lawrence

**Affiliations:** International Centre for Neurotherapeutics, Dublin City University, Collins Avenue, D09 V209 Dublin, Ireland; caren.antoniazzi@dcu.ie (C.A.); mariia.belinskaia2@mail.dcu.ie (M.B.); tom.zurawski@dcu.ie (T.Z.); seshukumar.kaza@dcu.ie (S.K.K.); oliver.dolly@dcu.ie (J.O.D.)

**Keywords:** botulinum neurotoxins, exocytosis, calcitonin gene-related peptide, migraine, nociception, trigeminal ganglion, capsaicin, SNAREs, SNAP-25, TRPV1

## Abstract

Chimeras of botulinum neurotoxin (BoNT) serotype A (/A) combined with /E protease might possess improved analgesic properties relative to either parent, due to inheriting the sensory neurotropism of the former with more extensive disabling of SNAP-25 from the latter. Hence, fusions of /E protease light chain (LC) to whole BoNT/A (LC/E-BoNT/A), and of the LC plus translocation domain (H_N_) of /E with the neuronal acceptor binding moiety (H_C_) of /A (BoNT/EA), created previously by gene recombination and expression in *E. coli*., were used. LC/E-BoNT/A (75 units/kg) injected into the whisker pad of rats seemed devoid of systemic toxicity, as reflected by an absence of weight loss, but inhibited the nocifensive behavior (grooming, freezing, and reduced mobility) induced by activating TRPV1 with capsaicin, injected at various days thereafter. No sex-related differences were observed. c-Fos expression was increased five-fold in the trigeminal nucleus caudalis ipsi-lateral to capsaicin injection, relative to the contra-lateral side and vehicle-treated controls, and this increase was virtually prevented by LC/E-BoNT/A. In vitro, LC/E-BoNT/A or /EA diminished CGRP exocytosis from rat neonate trigeminal ganglionic neurons stimulated with up to 1 µM capsaicin, whereas BoNT/A only substantially reduced the release in response to 0.1 µM or less of the stimulant, in accordance with the /E protease being known to prevent fusion of exocytotic vesicles.

## 1. Introduction

Serotypes A and E of botulinum neurotoxin (BoNT/A and /E), proteins (M_r_~150 k) produced by the requisite *Clostridium botulinum* and containing a disulphide-linked heavy (HC) and light chain (LC), are exquisite inhibitors of acetylcholine release from peripheral nerves [1]. Such preferential blockade underlies the great success of BoNT/A preparations in the clinical treatment of numerous conditions, due to over-activity of cholinergic nerves supplying various muscles or glands [2]. This selectivity and potent action are aided by the rapid exocytosis and recycling of small clear synaptic vesicles containing fast neurotransmitters [3], because their membrane possesses the high-affinity receptors for BoNT/A and /E, variants of synaptic vesicle protein 2 (SV2) [4,5,6]. A C-terminal domain of HC co-operatively binds gangliosides and the protein acceptor, a step shown to result in energy- and temperature-dependent endocytosis of BoNT/A at motor nerve terminals [7,8], which is accelerated by nerve stimulation [9]. Subsequent translocation to the cytosol has been attributed to the N-terminal portion of HC, whereas the metalloprotease activity of LC/A and /E, respectively, cleaves off 9 and 26 C-terminal residues from synaptosomal-associated protein with M_r_ = 25 k (SNAP-25), yielding SNAP-25_A_ or SNAP-25_E_ (reviewed by [10]). Such distinct proteolysis results in blockade of transmitter release because of their substrate being a SNARE (soluble N-ethylmaleimide-sensitive-factor attachment protein receptor), required for the exocytosis of all neurotransmitter types [11,12]. Accordingly, inhibition by BoNT/A of the stimulated release of pain mediators, such as substance P and calcitonin gene-related peptide (CGRP), established that the exocytosis of large dense-core vesicles from rat sensory neurons is susceptible to SNAP-25 cleavage [13,14,15,16,17]. Evidence for the involvement of CGRP in the pathology of migraine [18] has generated research interest in the possible potential of BoNT/A as an anti-nociceptive agent (see below).

Intraplantar pre-injection of BoNT/A complex was first reported to reduce peripheral inflammatory pain, along with alleviating nocifensive behavior in a rat model for formalin-induced pain [19]. Several studies in rodent models of mononeuropathy showed that BoNT/A administered in this way decreased allodynia in peripheral nerve constriction or ligation [20,21,22], ventral root transection [23], and infraorbital nerve constriction [24]. Moreover, prior administration of BoNT/A diminished the hyperalgesia and flare [25] induced by subcutaneous capsaicin, which activates the transient receptor potential vanilloid 1 (TRPV1), a pivotal transducer of pain signals [26,27]. Clinical investigations demonstrated the effectiveness of BoNT/A injected around the forehead in treating certain cases of chronic, but not episodic, migraine (reviewed by [28]). Based on the outcomes of large, double-blind randomized, placebo-controlled trials, which revealed a reduction in the number of migraine days [29,30,31], the FDA approved its use for chronic migraine in patients experiencing the symptoms for more than 15 days per month. The influence of the toxin on the frequency of migraine attacks varied between the trials, with a significant reduction obtained in some but not all patients [32,33].

CGRP levels in plasma are known to be increased during migraine attacks [34], and intravenous infusion of the peptide produces migraine-like symptoms in susceptible volunteers [35,36]. Antagonists (including antibodies [37,38]) of CGRP or its receptor usually alleviate headaches, although the outcomes of long-term blockade of CGRP signaling remain unknown [39]. Normalization of CGRP levels in cranial venous outflow can reduce pain [39,40]. BoNT/A has been found to lower the elevated amounts of CGRP in blood samples from some migraineurs [34], an action attributed to its inhibition of the release from sensory neurons. As this beneficial change was only observed in those that responded to the therapy, a search was warranted for a more efficacious variant of this neurotoxin. How to address this challenge was influenced by the expectation that a more extensive truncation of SNAP-25 with LC/E protease, rather than that of BoNT/A, would cause greater inhibition of CGRP release [17]. Due to BoNT/E proving unable to bind avidly and efficiently translocate into sensory neurons, addressing this relevant question necessitated recombinantly creating chimera BoNT/EA [41]. This consists of LC.H_N_/E (LC together with an N-terminal portion of the HC of BoNT/E) and H_C_/A (the acceptor binding C-terminal moiety of BoNT/A). As a further improvement, the short duration of action of /EA was extended by ligating the gene encoding LC/E to that for whole BoNT/A, whose di-leucine motif underlies its longevity. The resulting chimeric protein, LC/E-BoNT/A [42], displayed several advantages: (i) its H_C_/A constituent affords binding to the SV2C receptor on sensory neurons, which leads to translocation into the cytosol and predominant production of SNAP-25_E_; (ii) unlike BoNT/A, it blocks CGRP release from rat neonate trigeminal neurons (TGNs) in vitro when elicited by strong stimulation with 1 µM capsaicin; and (iii) ameliorates the nocifensive behavior in vivo arising from neuropathic pain in a rat spared nerve injury model [42].

In the present study, evidence was sought for the effectiveness of LC/E-BoNT/A on another type of pain, craniofacial acute nociception in rat. Moreover, the prospect of the two LC/E-containing chimeras offering any improvement as potential anti-nociceptives relative to BoNT/A was evaluated from their suppression of CGRP release from TGNs in vitro, when elicited by various concentrations of capsaicin, the stimulant used for the pain study.

## 2. Results

### 2.1. Injection of LC/E-BoNT/A into the Right Whisker Pad of Rats Does Not Alter Their Weight Gain, Grooming, Exploratory, or Locomotor Behavior

**Figure 1:**
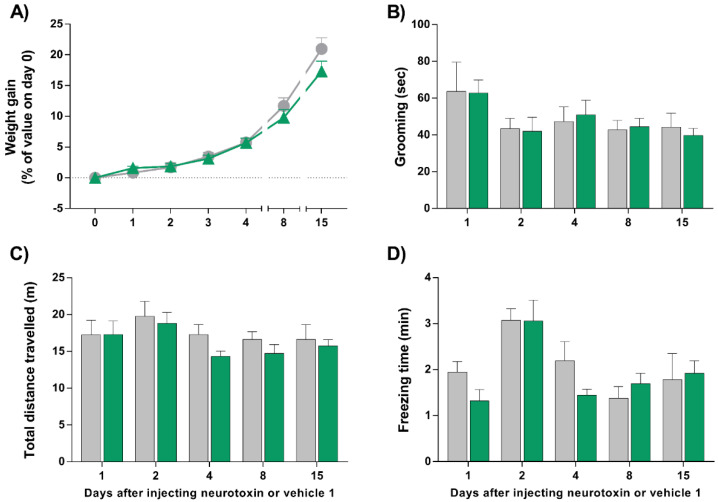
LC/E-BoNT/A injected into the right whisker pad does not cause weight loss or alter grooming and locomotor behavior in rats. (**A**) Weight gain after injection of the neurotoxin (75 units/kg) (▲) or vehicle 1 (●). (**B**) After administrating LC/E-BoNT/A (green bars) or vehicle 1 (grey bars), spontaneous grooming was quantified over 20 min as the time each animal spent rubbing the injected facial area with its paws, (**C**) locomotor behavior was assessed by the measurement of total distance moved, and (**D**) freezing time was observed. Data are expressed as mean + standard error of the mean (SEM) (n = 8) and were analyzed using Student’s *t*-test; no significant differences were found.

Single injections of LC/EBoNT/A (75 units/kg) into the right whisker pad of male and female rats had no impact on their weight gain (Figure 1A) or normal grooming behavior over the period studied (Figure 1B), when compared with the group that received vehicle 1 (neurotoxin-free control; see Section 5.). In addition, the exploratory activity was not affected by the neurotoxin injection, as the total distances travelled by both sets were comparable on all the days assessed (Figure 1C). Finally, immobility—another locomotor aspect regarded as an indirect marker of pain-like status and measured here by freezing time—was not significantly modified over time by LC/E-BoNT/A administration (Figure 1D). Hence, it can be concluded that, at the selected dose, LC/E-BoNT/A does not hinder animals’ natural behavior to a significant extent, and so it is valid to assess its effects in a capsaicin-induced rat model of acute nociception.

### 2.2. LC/E-BoNT/A Causes Long-Lasting Preventative Alleviation of Acute Nocifensive Behavior Induced by Capsaicin in Rats

**Figure 2:**
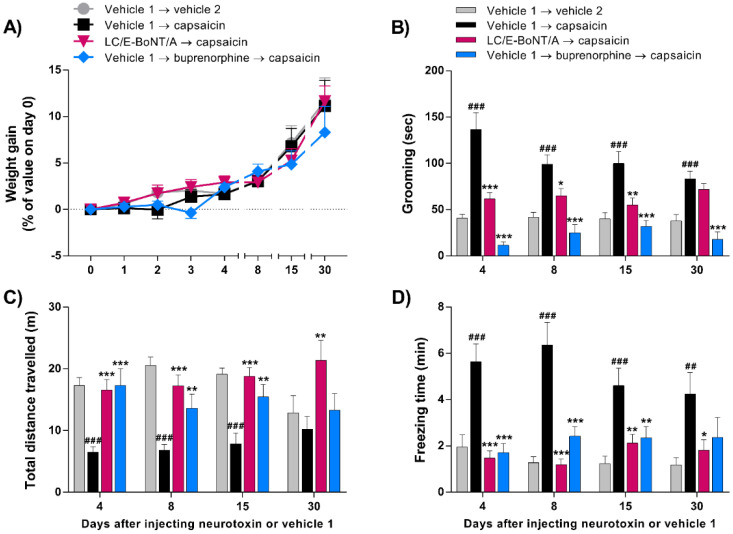
LC/E-BoNT/A showed a long-lasting prophylactic anti-nociceptive effect on acute nocifensive behavior induced by capsaicin, with the maximum amelioration observed on day 4 after neurotoxin injection into the right whisker pad. (**A**) Weight gained, (**B**) grooming, as the time each animal spent rubbing the injected facial area with its paws in a 20 min recorded period, (**C**) locomotor behavior assessed by the measurement of total distance walked, and (**D**) freezing time evoked by injection of capsaicin (2.5 µg in 20 µL) or vehicle 2 into rat right whisker pad, assessed at various days after administration of neurotoxin (75 units/kg). Data are expressed as mean + SEM (n = 10) and were analyzed by one-way ANOVA followed by Bonferroni’s post hoc test. ## *p* < 0.01, ### *p* < 0.001 vs. vehicle 1 → vehicle 2 group; * *p* < 0.05, ** *p* < 0.01, *** *p* < 0.001 vs. vehicle 1 → capsaicin group.

After verifying that LC/E-BoNT/A did not cause any discomfort or pain to the animals, as reflected by their unaltered natural behavior, another experimental cohort was injected with LC/E-BoNT/A (75 units/kg) or vehicle 1 into the right whisker pad, followed by vehicle 2 (see Section 5) or capsaicin (2.5 µg in 20 µL) on days 4, 8, 15, and 30 after pre-treatment with neurotoxin. As before, the neurotoxin did not affect weight gain compared to the other groups (Figure 2A). The most notable finding is that a single injection of LC/E-BoNT/A induced a long-lasting anti-nociceptive effect, as evidenced by a suppression of the acute nocifensive behavior evoked by capsaicin. The results revealed that in animals pre-injected with vehicle 1 (as a control for neurotoxin), the subsequent administration of capsaicin triggered nocifensive behavior. This was manifested by a significant increase in grooming (Figure 2B) and freezing time (Figure 2D), while it decreased the distance (Figure 2C) walked in the testing cage, compared to vehicle 1 → vehicle 2-injected control group. On the other hand, rats pre-treated with LC/E-BoNT/A showed a significant reduction in grooming behavior after injecting capsaicin on days 4, 8, and 15 (Figure 2B); after 30 days, no significant effect was apparent. As a positive control, the subcutaneous injection of the opioid analgesic buprenorphine (0.2 mg/kg) 30 min before capsaicin prevented the grooming intensification (Figure 2B), verifying the latter as a nocifensive behavior. Locomotor activity was substantially decreased by capsaicin, reflected in shorter total distances walked on days 4, 8, and 15, when compared to the vehicle 2-treated control group; this change was reversed to a major extent at various times in the animals pre-treated with the neurotoxin (Figure 2C). As noted above, the freezing time was also significantly increased after capsaicin administration in comparison with the vehicle 2 controls. Notably, in rats pre-treated with LC/E-BoNT/A this pain-like effect was completely reversed on days 4 and 8 and, to a lesser extent, on the subsequent days (Figure 2D). As expected, administration of buprenorphine also prevented the impairments in the locomotor activity evoked by capsaicin (Figure 2C,D).

### 2.3. LC/E-BoNT/A Equally Diminishes Nocifensive Behavior Evoked by Capsaicin in Both Male and Female Rats

**Figure 3:**
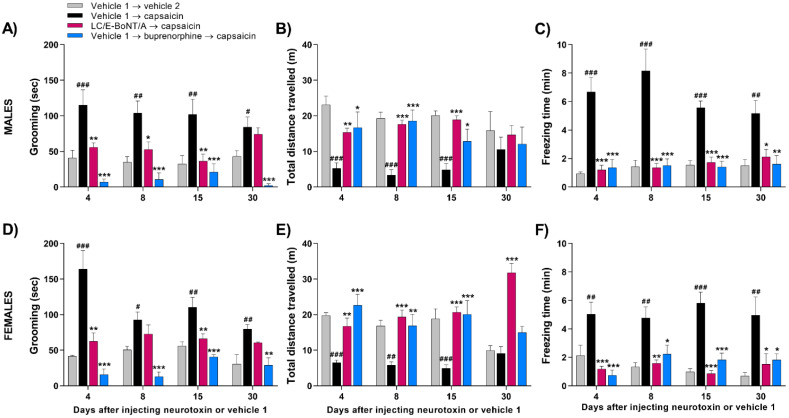
LC/E-BoNT/A exerted a similar long-lasting anti-nociceptive effect in both male and female rats on the acute nocifensive behavior evoked by capsaicin. Grooming behavior in males (**A**) and females (**D**), locomotor activity in males (**B**) and females (**E**), and freezing time for males (**C**) and females (**F**) evoked by injection of capsaicin (2.5 µg in 20 µL) or vehicle 2 into right whisker pad, evaluated at various days after administration of the neurotoxin (75 units/kg) or vehicle 1. Data are expressed as mean + SEM (n = 4/group in vehicle 1 → vehicle 2 and vehicle 1 → buprenorphine → capsaicin; n = 5/group in vehicle 1 → capsaicin and LC/E-BoNT/A → capsaicin) and were analyzed by one-way ANOVA followed by Bonferroni’s post hoc test. # *p* < 0.05, ## *p* < 0.01, ### *p* < 0.001 vs. vehicle 1 → vehicle 2 group; * *p* < 0.05, ** *p* < 0.01, *** *p* < 0.001 vs. vehicle 1 → capsaicin group.

Evidence was sought for similarities or differences in the effectiveness of LC/E-BoNT/A in both sexes for lowering the capsaicin-induced increase in the acute nociceptive behavior. Another experimental cohort including male and female rats was injected with LC/E-BoNT/A into the right whisker pad, and the acute nociceptive behavior evoked by capsaicin was assessed as above. Again, LC/E-BoNT/A did not influence the weight gain in either sex (data not shown). Capsaicin administration evoked the nocifensive response indicated by increased grooming (Figure 3A,D), accompanied by a decrease in the exploratory and locomotor behavior in males (Figure 3B,C) and females (Figure 3E,F) previously injected with vehicle 1. Although females appeared more responsive to capsaicin on day 4, there was no significant difference from males, so this likely reflects random variation. For both sexes, the substantial influence of LC/E-BoNT/A on decreasing grooming occurred predominantly on days 4 and 15, with no significant change on day 30 (Figure 3A,D). In each case, the capsaicin-induced reduction in total distance walked was diminished by pre-treatment with the neurotoxin (Figure 3B,E), and the immobility (reflected by freezing) that resulted from capsaicin administration was considerably reduced in rats pre-treated with LC/E-BoNT/A compared to the vehicle 1 → capsaicin-injected controls (Figure 3C,F). Additionally, administration of buprenorphine prevented the elevation of grooming and impairments in locomotor activity evoked by capsaicin in both sets of rats (Figure 3A–F).

### 2.4. LC/E-BoNT/A Precludes the Induction of c-Fos Expression in the Trigeminal Nucleus caudalis (TNC) after Capsaicin Injection into the Whisker Pad; a Biochemical Indication of Reduced Nociceptor Activation

**Figure 4:**
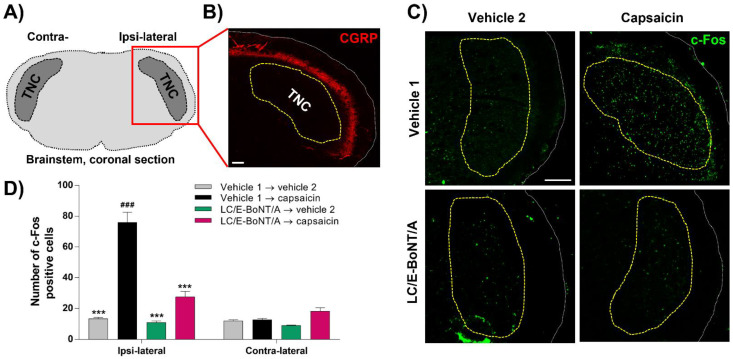
LC/E-BoNT/A prevents nociceptive neural activity in trigeminal nucleus caudalis (TNC), induced by capsaicin injected into the right whisker pad of rats, 4 days after neurotoxin administration. (**A**) Schematic diagram of the brainstem coronal section, showing the location of TNC. (**B**) Calcitonin gene-related peptide (CGRP) immuno-staining (red) was used to identify the outer border of the TNC area (yellow line). (**C**) Representative fluorescent images of c-Fos expression in TNC ipsi-lateral injected side; the green dots represent the c-Fos-positive cells. (**D**) These were quantified in the TNC region (determined as in B, but note that the CGRP staining is not shown in **C**), in 4 randomly-selected sections per animal. Data are expressed as mean + SEM (n = 3 animals per group). Data were analyzed using two-way ANOVA followed by Bonferroni’s post hoc test. ### *p* < 0.001 vs. vehicle 1 → capsaicin contra-lateral group; *** *p* < 0.001 vs. vehicle 1 → capsaicin ipsi-lateral group. Scale bars: 100 µm.

The effect of this neurotoxin on the neural activation evoked by capsaicin was assessed by quantifying the expression of c-Fos in the TNC in the brainstem of rats (Figure 4A); this was relevant because afferents from the whisker pad project into the TNC. As the inhibition of nocifensive behavior was observed 4 days after LC/E-BoNT/A administration into the right whisker pad, samples were collected at this time for c-Fos detection. The TNC is highlighted by the dashed yellow line (Figure 4B,C), whose outer border was delineated by CGRP staining (red) (Figure 4B). The number of c-Fos-positive cells found in TNC was significantly higher in ipsi-lateral sections from the group injected with capsaicin, compared to the ipsi-lateral vehicle 2-injected (control) and contra-lateral vehicle 1 → capsaicin groups (Figure 4C,D). Consistent with the neurotoxin’s alleviation of nocifensive behavior, the animals pre-treated with a single injection of LC/E-BoNT/A showed a substantial reduction in the number of cells expressing c-Fos in the ipsi-lateral side where capsaicin was injected, with a 77 ± 4% inhibition of this marker on day 4 after administration of the neurotoxin. In contrast, no alterations were observed in the c-Fos expression in cells residing on the contra-lateral side to the injections (Figure 4D).

### 2.5. CGRP Release from TGNs Evoked by Strong Stimulation Is Diminished by LC/E-BoNT/A or BoNT/EA, whereas BoNT/A Only Reduces the Response to Lower Capsaicin Concentrations

**Figure 5:**
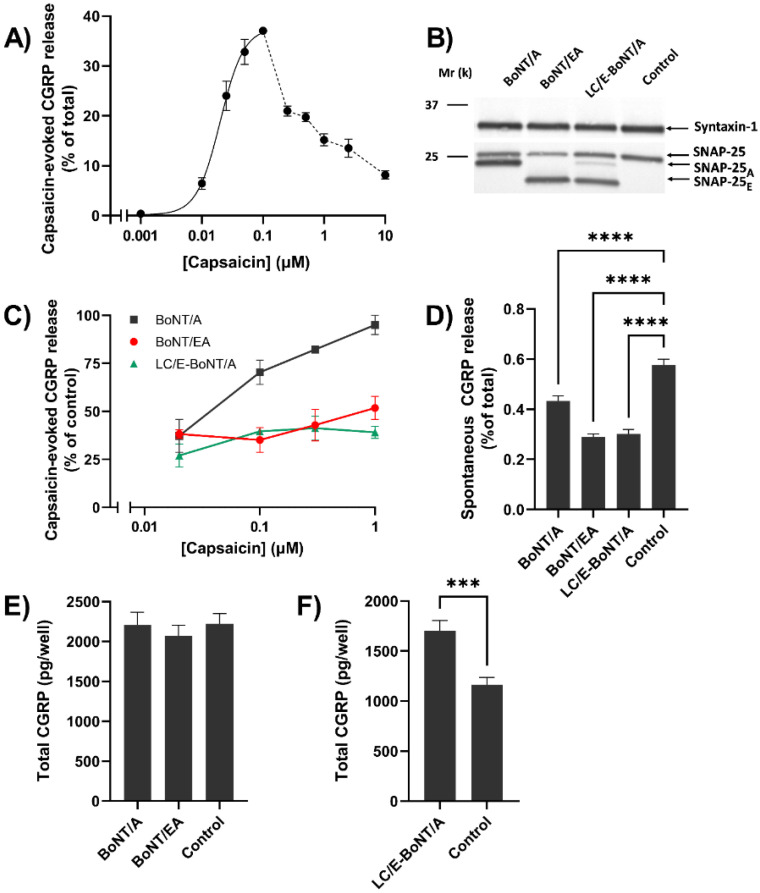
Capsaicin concentration-dependency for stimulation of CGRP exocytosis from trigeminal ganglion neurons (TGNs) and its inhibition by BoNT/A, BoNT/EA, and LC/E-BoNT/A; only the two latter SNAP-25_E_-producing variants retained efficacy against high capsaicin concentration, despite cleaving no more SNAP-25 than BoNT/A. (**A**) TGNs were exposed to various capsaicin concentrations ([CAP]) for 30 min, and the amount of CGRP released into the bathing solution was quantified and expressed as a % of the total CGRP content (i.e., amount released plus quantity retained inside the cells). The inclining part of the relationship (0.001 to 0.1 μM) was fit with a four-parameter logistic function with R^2^ = 0.884, yielding a half-maximal effective [CAP] (EC_50_) = 0.02 μM. Individual values for the declining part (0.1 to 10 μM) are connected by a broken line. (**B**) Western blot of detergent-solubilized lysates of cells that had been pre-incubated for 48 h in the absence (control) or presence of 100 nM of the indicated BoNTs, using an antibody recognizing intact SNAP-25 and cleavage products of BoNT/A (SNAP-25_A_) or chimeras containing /E protease (SNAP-25_E_). The blot was additionally probed with an antibody to syntaxin-1A/B. Black lines to the left indicate the migration of 37 and 25 kDa molecular weight standards. (**C**) The amounts of CGRP released, evoked during 30 min by [CAP] from TGNs pre-treated with the toxins as above, are expressed as % of requisite control values for this experiment (7.3 ± 1.2, 33.6 ± 2.8, 24 ± 3.3, and 13.3 ± 2.1% of total CGRP for 0.02, 0.1, 0.3 and 1 μM CAP, respectively). (**D**) Spontaneous CGRP release over 30 min exposure to HEPES buffered saline lacking capsaicin. (**E**,**F**) The mean amounts of total CGRP in cells treated as above. As a lower seeding density of cells was used for the experiments in (**F**) than in (**E**), smaller CGRP amounts were detected in the former. Data are presented as mean + SEM. For (**D**,**E**), one-way ANOVA was used followed by Bonferroni’s post hoc test, and significance of the latter is indicated with asterisks; **** *p* < 0.0001, n ≥ 16, N ≥ 4. For (**F**) unpaired two-tailed *t*-test with Welch correction was used, *** *p* < 0.001, n = 15, N = 2.

In view of the variable success of migraine treatment with BoNT/A (see Section 1), the ability of this protease to block the CGRP release evoked by activating TRPV1 to different extents with a range of capsaicin concentrations was examined using rat TGNs in culture. The amounts of peptide exocytosed and retained by the cells, respectively, was quantified by enzyme-linked immunosorbent assay (ELISA). A concentration-dependent increase in release was obtained with 0.001–0.1 µM of the vanilloid, representing 0.5–37% of total CGRP (Figure 5A), but lower levels were seen with 0.25–10 µM (see Section 3). For assaying susceptibility of the release to BoNT/A, the cultured neurons were incubated with 100 nM for 48 h, so that the bulk (75%) of their total SNAP-25 content was truncated (Figure 5B); consistent with the neurotoxin’s substrate specificity, syntaxin 1 remained unaffected, as revealed by the Western blotting (Figure 5B). Nevertheless, such extensive pre-treatment with BoNT/A failed to prevent CGRP exocytosis elicited by 1 µM capsaicin (Figure 5C). However, upon lowering the amounts of capsaicin used for the stimulation, the release of CGRP became progressively inhibited (Figure 5C), though incompletely (see Section 3). To examine the possibility of BoNT/A-cleaved SNAP-25 (SNAP-25_A_) mediating exocytosis when evoked by the high intracellular Ca^2+^ concentration ([Ca^2+^]_i_) known to be induced by the larger capsaicin concentrations, TGNs were incubated for 48 h with 100 nM chimeric LC/E-BoNT/A or BoNT/EA; these truncated a similar majority (63 and 77%) of their common target to SNAP-25_E_ (Figure 5B). Under these two conditions, the pronounced reduction in CGRP release elicited by all of the capsaicin concentrations was maintained (Figure 5C). It is noteworthy that each of the neurotoxins caused a limited, but significant, suppression of the spontaneous release of the peptide (Figure 5D). The total cellular content of CGRP was increased somewhat by BoNT/A or BoNT/EA, but this change only reached significance for cells treated with LC/E-BoNT/A (Figure 5E); this probably arose from decreased resting release during the long pre-incubation with the neurotoxins.

## 3. Discussion

Much pain research is being focused on migraine and trigeminal neuralgia, because of the prevalence of these debilitating conditions that involve the trigeminal sensory system. It conveys sensory information from the craniofacial region, being composed of peripheral structures such as the trigeminal nerve and associated ganglia, as well as central structures such as the dorsal brainstem region, which includes the TNC (reviewed by [43]). Sensory inputs from the periphery are relayed by afferent fibers that make connections with second-order neurons in the TNC, so information gets propagated to the thalamus where sensory stimuli are processed. Third-order neuronal projections conduct the stimulus to the somatosensory cortex and insula; there, signals are interpreted with respect to location, intensity, and duration [44,45]. Considering that one of the three branches of the trigeminal nerve, the infraorbital branch, innervates the rats’ whisker pad, this accessible site was preferred herein for administering a TRPV1 agonist, capsaicin. The objective was to induce acute nociception, manifested by increased animal attention to the injected area as reflected by intensification of grooming [46]. This allowed evaluation of the anti-nociceptive versatility of LC/E-BoNT/A, as shown previously in a neuropathic pain model [42]. Acute nociception is deemed an attractive, lesion-free system for unveiling meaningful and reliable benefits of new analgesics [47]. This investigation initially examined spontaneous behavior, including locomotor activity, grooming, freezing, as well as body weight. Follow-on experiments utilized capsaicin as a means to trigger acute pain, because it recruits pathophysiological mechanisms distinct from those involved in neuropathic models [48], as referred to above. In fact, it sensitizes peripheral and central nociceptive circuits underlying the manifestation of nociceptor sensitization, thereby, representing a more generalized approach than those provided by modelling aspects of disease [47]. The expected acute nociception caused by capsaicin is reflected by significantly increased grooming, freezing, and reduced movement, at virtually all time points relative to the requisite controls (Figure 2 and Figure 3). The variation in these values seen with the groups of rats at different days made it difficult to attribute significance to changes in response following repeated exposure to capsaicin. These arise, at least in part, because the first exposure to capsaicin produces a heightened response, due to an aversive novelty associated with the environment, and this tends to be attenuated upon repeated testing [49]. Finally, it is reassuring that buprenorphine reversed the effects of capsaicin on all of the parameters measured, considering that this semi-synthetic opioid primarily causes partial agonism of the mµ opioid receptor (for review, see [50]).

Advantageously, it emerged that it is valid to exploit this experimental system for evaluating the effect of LC/E-BoNT/A on evoked nociception, because its unilateral administration into one whisker pad did not modify any of the measured parameters of unprovoked behavior. In the case of grooming, the significant reduction of the response to capsaicin observed after pre-treatment with LC/E-BoNT/A persisted up to day 15, and this approximates to its amelioration of nocifensive behavior in a spared nerve injury model of neuropathic pain [42]. A similar pattern of decreases in capsaicin-induced freezing by the neurotoxin was seen, except some relief was apparent after 1 month, though at a lower level of significance. Likewise, the reduction of locomotor activity induced by capsaicin was improved significantly by LC/E-BoNT/A. As it is well established and accepted that these three parameters are usually altered when animals experience painful events (reviewed in [51]), the changes resulting from this treatment are indicative of an analgesic influence of LC/E-BoNT/A. Encouragingly, undesirable neurotoxic effects can be excluded from the protein’s benefits, because weight gain—used as a reliable indicator of BoNT toxicity [52]—was not impaired by the low dose of LC/E-BoNT/A that proved effective. A comparison with the efficiency of BoNT/A on nociception induced by peripherally-applied capsaicin in rodents is conveniently afforded by published data [25,53,54]. Using the foot pad as the locus for injections, both the mechanical and thermal hyper-sensitivity induced by capsaicin were reported to be alleviated by BoNT/A 6 days after administration [53], close to the interval of 4 days that yielded the maximum improvement with LC/E-BoNT/A. At 7 days after BoNT/A application to the rat whisker pad, the increased grooming resulting from capsaicin was reported to be prevented [25]. It is also notable that equivalent levels of relief of nocifensive behavior were demonstrated with BoNT/A over similar periods of 7–21 days in mice, though using a different peripheral injection site [54]. Critical importance is being attached to studying pain therapeutics in females, as well as the more commonly used males [55], because of their greater sensitivity to pain and lower responsiveness to analgesics [56,57]. Despite some variability in the times females spent grooming after capsaicin application, this parameter was extensively suppressed by LC/E-BoNT/A. Likewise, although the total difference travelled by subjects also varied on different test days, capsaicin clearly reduced mobility on days 4, 8, and 15; there were no significant or systematic differences in the outcomes for LC/E-BoNT/A on these days or between sexes.

As the TNC contributes to the transmission of craniofacial pain, acute noxious stimulation of the trigeminal innervation induces the expression of c-Fos in the nuclei of neuronal cell bodies within this region; thus, it serves as a marker of nociception [58,59]. After unilateral whisker pad administration of LC/E-BoNT/A and later evoking pain with capsaicin, this indicator was monitored in both TNCs, ipsi- and contra-lateral to the injections. Capsaicin caused a five-fold increase in c-Fos expression, only in the ipsi-lateral side, in keeping with its elevation of nociception being restricted to this locus. This finding accords with the outcomes of earlier studies using peripheral capsaicin [60,61] or other inflammatory agents, such as formalin [62,63,64], and complete Freud’s adjuvant [65]. Importantly, the elevation in c-Fos expression resulting from capsaicin was significantly lowered with the LC/E-BoNT/A pre-treatment (Figure 4C,D). In contrast, the neurotoxin did not exert any significant change in the c-Fos values for the contra-lateral side or when capsaicin was omitted. This new finding correlates with the attenuation by LC/E-BoNT/A of pain-like behavior discussed above, substantiating the evidence for consequential activity in ipsi-lateral TNC after peripheral application. Such an outcome is in line with a report of a similar influence on formalin-induced c-Fos-like immuno-reactivity in the TNC [62,63]. Reduced c-Fos denotes decreased activity at central terminals, which could arise from LC/E-BoNT/A affecting the peripheral terminals and consequently suppressing the activity of the primary nociceptors. On the other hand, there is now extensive evidence that at least some fraction of the BoNT/A that enters nociceptors after peripheral injection undergoes retrograde axonal transport to the central nervous system (reviewed by [10,66,67]). With specific regard to the trigeminal system and the possible mechanism of action of BoNT/A alleviating migraine symptoms, a contribution of peripheral blockade of neurotransmitter release from sensory neurons is generally accepted, but widely considered unable to explain all the benefits accredited to clinical treatment with this toxin [67]. However, this may have to be re-appraised due to the emerging success of migraine treatment with subcutaneous injections of CGRP sequestering monoclonal antibodies [38]. By contrast, the precise central locus (or loci) of BoNT/A action, details of the processes inhibited, and the relative contribution of each to analgesia are considered speculative [67]. Proposed sites of action include the trigeminal ganglia, where the somata of most primary cranio-facial nociceptors reside, the pre-synaptic central terminals of primary nociceptors in the TNC, the post-synaptic site of these same junctions after trans-synaptic transfer of BoNT/A (or its protease) to the second-order central nociceptors, or even sites in higher order neurons after further intra-axonal transport along ascending nociceptive pathways. At any single one (or combination) of these site(s), it is speculated [67] that BoNT/A may inhibit chemical neurotransmission and/or the insertion of signaling proteins into neuronal cell membranes, to interfere with the passage of noxious signals towards pain processing centers in the brain.

Research attention was also devoted in this study to the stimulation of CGRP release from TGNs by capsaicin, a widely-used agonist of a non-selective cation channel, TRPV1, that is a key transducer of sensory signals [68]. The bell-shaped dose–response curve obtained showed that 10–100 nM capsaicin gave an expected increase in Ca^2+^-dependent exocytosis of the pain-mediating peptide, consistent with activation of TRPV1 leading to Ca^2+^ influx through its ion pore. This accords with the concentration-dependent elevation by capsaicin of [Ca^2+^]_i_ observed in cultured dorsal root ganglia (DRG) neurons [69] and in trigeminal ganglia or DRG explants of *pirt*-GCaMP3 mice [70,71]. However, the substantially lower level of CGRP released with the larger capsaicin concentrations (0.25–10 µM), despite the likelihood of continued dose-dependent increases in [Ca^2+^]_i_ [69] seems to be suggestive of a downregulation, in which the exocytotic process becomes refractory to high [Ca^2+^]_i_. Indeed, an equivalent effect is apparent in adrenal chromaffin cells permeabilized by electrical discharges; lower amounts of catecholamines were released from those permeabilized in the presence of 1 mM Ca^2+^ compared to lesser concentrations [72].

Another intriguing observation is that the lower quantities of CGRP-release evoked by 1 µM capsaicin could not be eradicated by an extensive pre-treatment of TGNs with BoNT/A, which truncated a majority of its target to SNAP-25_A_ (Figure 5B). Notably, reducing the capsaicin concentrations used led to the onset of inhibition of the response (Figure 5C). It is noteworthy that abolition of CGRP release evoked by all capsaicin concentrations tested was not achieved by any of the three neurotoxins; this likely relates to an appreciable proportion of SNAP-25 remaining intact, despite prolonged pre-exposure to a high concentration of either chimera or BoNT/A; the reasons for this remain unclear. The ineffectiveness of BoNT/A in blocking the release triggered by the higher concentrations of stimulant could be explained by the known ability of 1 µM capsaicin to cause a more rapid, prolonged, and higher elevation of [Ca^2+^]_i_ in TGNs [17]; this may allow exocytosis under such exceptional circumstances to be mediated by SNAP-25_A_ (see below). Furthermore, it has been reported that BoNT/A does not prevent the increased exocytosis of an intra-vesicular domain of synaptotagmin I when this vanilloid triggers SNARE-dependent vesicle recycling in TGNs [17]. Notably, this proposal explains why raising [Ca^2+^]_i_ with an ionophore reverses the inhibition of transmitter release from both TGNs and motor nerves [17,73,74], and is compatible with SNAP-25_A_ forming SDS-resistant, stable complexes with the other SNARE partners required for exocytosis [17,73,75] that can be disassembled by N-ethylmaleimide sensitive-factor [76]. Such an explanation is strengthened further by the demonstration that deleting 26 residues from SNAP-25 with LC/E-BoNT/A or BoNT/EA diminished the CGRP release elicited by all the capsaicin concentrations (Figure 5C); likewise, /EA prevents vesicle exocytosis induced by 1 µM of the stimulus, as revealed previously by the Syt-Ecto assay [17]. Moreover, electrophysiological recordings in brain stem slices containing sensory neurons revealed that BoNT/EA, unlike /A, eliminates the excitatory effects of CGRP that result from capsaicin activating TRPV1 [17]. Finally, the release of CGRP evoked from TGNs with 1 µM capsaicin is known to be inhibited by a chimera composed of LC/E attached to a protease-inactive mutant of BoNT/A, termed LC/E-BoTIM/A [77]. In short, it is clear that this CGRP exocytosis elicited by the high concentrations of the TRPV1 activator cannot be mediated by SNAP-25_E_, and accords with the elegant demonstration that BoNT/A slows exocytosis from vesicles following their fusion, especially at high [Ca^2+^]_i_; whereas, BoNT/E acts at an earlier stage to prevent complex formation and transmitter release [78,79].

## 4. Conclusions

LC/E-BoNT/A significantly reduced capsaicin-induced acute nociception over several days, after unilateral administration into a whisker pad, without altering spontaneous behavior or locomotor performance in control rats. Its profound inhibition of CGRP release from sensory neurons in vitro, even when intensely stimulated with capsaicin, raises the possibility of this chimera proving more effective against painful conditions poorly responsive to BoNT/A.

## 5. Materials and Methods

### 5.1. Materials

Capsaicin and nerve growth factor (NGF) 2.5S were purchased from Alomone Labs (Jerusalem, Israel) and buprenorphine from Dechra Ltd. (Lostock Gralam, Staffordshire, UK). Culture 48-well plates were purchased from Thermo Fisher (Cheshire, UK). Collagenase and Dispase^®^ were supplied by Bio-Sciences (Dún Laoghaire, Co. Dublin, Ireland). Monoclonal antibodies specific for SNAP-25 plus its BoNT/A- and /E-cleavage products (SMI-81) and syntaxin-1A/B (S0664) were purchased, respectively, from Covance (now Labcorp Drug Development, Princeton NJ, USA) and Merck (Arklow, Co. Wicklow, Ireland). A rabbit polyclonal antibodies against c-Fos (ABE 457) were bought from Merck, whilst Abcam (Cambridge, Cambs., UK) supplied a mouse monoclonal antibody specific for CGRP (ab81887). Donkey secondary antibodies reactive with rabbit or mouse IgGs and labelled with Alexa Fluor 488 or 555, respectively, (A21206 and A31572) were provided by Invitrogen (Fisher Scientific, Loughborough, Leics., UK), also the supplier of Prolong™ Glass Anti-fade Mountant. Anti-mouse alkaline phosphatase (AP)-conjugated secondary antibodies (A3688) were purchased from Merck. Western blotting reagents: polyvinylidene fluoride membrane (PVDF) and Bio-Rad protein standards were bought from Fannin Healthcare (Leopardstown, Co. Dublin, Ireland). Lithium dodecyl sulphate (LDS) sample buffer and 12% BOLT™ Bis-Tris polyacrylamide gels were from Bio-Sciences. ELISA kits were purchased from Bertin Technologies (Montignyle Le Bretonneux, Île-de-France, France). All other reagents were obtained from Merck, unless otherwise specified.

### 5.2. Animals

This project was approved on 1 May 2018 by the Research Ethics Committee of Dublin City University (DCUREC/2018/091), Ireland, following the University’s policy on the use of animals. The animal husbandry and all associated scientific procedures were authorized by the Health Products Regulatory Authority of Ireland (Project Authorization no. AE19115/P020 approved on 5 October 2018), under the European Union (Protection of Animals used for Scientific Purposes) Regulations 2012 (S.I. No. 543 of 2012), in accordance with Directive 2010/63/EU of the European Parliament and of the Council of 22 September 2010 on the protection of animals used for scientific purposes. Eighty-four adult Sprague–Dawley rats, including males and females (weight 210–290 g), purchased from Charles River Laboratories (Margate, Kent, UK), were used for this study. They were housed in Tecniplast™ Double-Decker Sealsafe^®^ Plus cages, individually ventilated, at a stocking density not exceeding 5 per cage. Rats were bedded on sawdust, supplied with nesting material and kept under a constant 12 h/12 h light/dark cycle with free access to food and water. The animals were acclimatized for at least one week before experiments, their weights were monitored and recorded daily. Behavioral studies and tissue collection were carried out according to guidelines for animal research reporting in vivo experiments (ARRIVE 2.0) [80].

### 5.3. Drugs and Treatments

LC/E-BoNT/A was expressed recombinantly in *E. coli* and purified, using a modification of the procedures previously described [42]. Its biological activity was confirmed by a mouse lethality assay, yielding a specific neurotoxicity of 6 (±1) × 10^7^ mouse medium lethal dose (mLD_50_) units/mg [81]. BoNT/A and /EA were also expressed in *E. coli* according to previously published procedures [41,77], yielding proteins with specific neurotoxicity of 2 × 10^8^ and 7 × 10^6^ mLD_50_ units/mg, respectively.

For the initial experiments, to ascertain if LC/E-BoNT/A, per se, influences the natural spontaneous behavior of male and female rats, the animals were assigned to two groups: those injected with vehicle 1 (0.05% human serum albumin in 0.9% NaCl) or LC/E-BoNT/A in the latter solution. In the subsequent sets, the animals were randomly assigned to 5 different treatments involving sequential injections: vehicle 1 → vehicle 2 (5% ethanol/5% Tween 80/0.9% NaCl); vehicle 1 → capsaicin; LC/E-BoNT/A → vehicle 2; LC/E-BoNT/A → capsaicin, and vehicle 1 → buprenorphine → capsaicin. Before injection, capsaicin was freshly dissolved (125 µg/mL) in vehicle 2. All behavioral assessments were performed between 11.00 and 18.00 by an operator unaware of the treatments given to the animals. The rats were anaesthetized with 3.5% isoflurane and given a unilateral single subcutaneous injection of 30 µL of LC/E-BoNT/A (75 units/kg) into their right whisker pad (perinasal area), using a Hamilton syringe (50 µL) fitted with a 30-gauge needle. Controls received 30 µL of vehicle 1. The animals were then returned to their home cages and left undisturbed until testing, except for daily monitoring of weight, motor, and physiological status. For the next experiments, the rats were first injected with the neurotoxin or its vehicle and starting 4 days later were given capsaicin or its vehicle, as detailed in Section 5.4. The final series involved subcutaneous administration of buprenorphine (0.2 mg/kg), an opioid modulator with strong anti-nociceptive effects, as a positive control, followed 30 min later by capsaicin.

### 5.4. Behavioral Assessments and Capsaicin-Induced Pain-Related Response (Acute Nociception)

The behavioral testing was performed in a quiet room with a temperature of 20 ± 1 °C, at days 1, 2, 4, 8, 15, and 30 after injection of the neurotoxin or vehicle 1. Capsaicin-induced acute nociception was tested on days 4, 8, 15, and 30 after injecting LC/E-BoNT/A or vehicle 1 into the different groups of rats; times were chosen based on previous experiments performed in our laboratory [42]. Ten minutes before behavioral testing, rats were taken to the observation room and individually placed in transparent acrylic cages (50 × 30 × 25 cm) for acclimatization. Then, they were briefly restrained and injected with capsaicin (2.5 µg/20 µL with a 30 G needle fitted to a Hamilton syringe) or vehicle 2 into the right whisker pad. Immediately after injection, the rats were placed into a recording cage and the behavior was video recorded for 20 min. The length of recording was selected based on pilot studies performed previously. The acute nocifensive response was taken as the cumulative amount of time each animal spent grooming (face-wash strokes, chin/cheek rubs, hind paw face scratching) the injected facial area [82]. The recorded videos were analyzed by an observer blinded to the experimental conditions. Additional data extracted from the recordings, such as the total distance walked (meters) in the arena and the freezing time (minutes), were assessed by using the ToxTrac^®^ software (version 2.91, Universidade Da Coruña).

### 5.5. Collection and Fixation of Tissue

The animal tissues were processed at 4 days after LC/E-BoNT/A administration into the right whisker pad, due to the maximum inhibition of nocifensive behavior being observed then. Briefly, 2 h after completion of behavioral testing, the rats were over-dosed with pentobarbital sodium (Euthatal^®^, 200 mg/kg, intraperitoneal injection) and transcardially perfused through the ascending aorta with 200 mL of heparinized 0.9% NaCl, followed by fixation with 150 mL of 4% paraformaldehyde in 0.1 M sodium phosphate buffer pH 7.4 (PB). The brainstem was dissected and the area containing the TNC was post-fixed using the same solution for up to 4 h at room temperature. Tissues were then immersed in PB containing 15% sucrose at 4 °C overnight, followed by transfer to 30% sucrose in PB the next day and kept until the tissue sank. The samples were then removed from sucrose and frozen using isopentane (2-methyl butane), cooled in liquid nitrogen, and immediately stored at −80 °C.

### 5.6. Immuno-Histochemistry

The effects of LC/E-BoNT/A on neural activation evoked by capsaicin were assessed by quantifying the expression of c-Fos in the TNC of animals injected with 75 units/kg LC/E-BoNT/A or vehicle 1 into the right whisker pad; 4 days later, capsaicin (2.5 µg/20 µL) was administered as above (Section 5.4). The caudal brainstem containing the TNC, identified from atlas plates 135–158 [83], was embedded in O.C.T compound (Tissue-Tek, Sakura Finetek, Tokyo, Japan), cryo-sectioned (40 µm thick coronal sections) using a Leica CM3050 S cryostat (Leica Biosystems, Milton Keynes, Bucks, UK), and collected for free-floating in PB containing 0.9% NaCl (PBS). Two consecutive sections were placed in one well of a 48-well plate, and then the next 2 sections in an adjacent well; this process was continued until all sections were collected. These were rinsed thrice for 5 min with fresh PBS containing 0.1% Triton X-100 (PBST) before blocking non-specific immuno-reactivity by incubating the samples with 5% normal donkey serum (NDS) in PBST for 1 h. Then, the slices were incubated overnight at 4 °C with a rabbit polyclonal anti-c-Fos antibody (1:500 dilution in PBST + 5% NDS) and co-stained with a mouse monoclonal anti-CGRP antibody (1:1000 dilution in PBST + 5% NDS). The next day, after rinsing (3× for 5 min) with PBST, the slices were incubated for 2 h in the dark at room temperature with donkey anti-rabbit Alexa Fluor 488 and anti-mouse Alexa Fluor 555-labelled secondary antibodies, diluted 1:1000 in PBST. Then, sections were washed (3× for 5 min) with PBST, mounted on glass slides with ProLong™ Glass Antifade Mountant, and visualized with a confocal microscope (LSM 710; Carl Zeiss, Oberkochen, BW, Germany). Argon and helium/neon lasers provided the 488 nm and 543 nm lines for excitation of Alexa Fluor 488 and 555, respectively, with images acquired through an EC Plan-Neofluar 10 × /0.30 NA objective using Zen 2011 software (Carl Zeiss). Omission of secondary antibodies was used as a negative control. The number of c-Fos fluorescently-labelled cells was counted using ImageJ software (ImageJ 1.53e, National Institutes of Health, Bethedsa, MD, USA) in ipsi- and contra-lateral sides within observable borders of the TNC that were delineated by CGRP staining. The colored micrographs were converted to 8-bit grayscale TIFF images, the same threshold was applied to all images and positive nuclei were counted manually, using the multi-point tool. The average number of positive cells was calculated using 4 randomly-selected sections from groups of 3 animals for each treatment.

### 5.7. Isolation and Culturing of Rat Neonate TGNs

Trigeminal ganglia were dissected from 3 to 6 day-old Sprague–Dawley rat neonates, as described in [16], and kept in ice-cold Ca^2+^/Mg^2+^- free Hank’s balanced salt solution. After digestion with 1:1 (*v*/*v*) mixture containing 1275 U collagenase I and 17.6 U Dispase^®^ for 30 min at 37 °C, 12.5 U of Benzonase^®^ nuclease was added to reduce viscosity and clumping of the tissue; cells were gently triturated with a 2.5 mL Pasteur pipette and incubated at 37 °C for another 15 min. Then, the dissociated cell suspension was centrifuged through a discontinuous Percoll^®^ gradient, as described in [84], to separate neurons from non-neuronal cells, myelin, and nerve debris, before re-suspension in Dulbecco’s Modified Eagle Medium containing 10% (*v*/*v*) fetal bovine serum, 1% (*v*/*v*) penicillin-streptomycin, B-27^TM^ Supplement, and 50 ng/mL 2.5S NGF. The resultant TGNs were seeded at a density of ~30,000 neurons per well in 48-well plates that had been pre-coated with poly-L-lysine (0.1 mg/mL) and laminin (10 µg/mL). To suppress the growth of dividing (i.e., non-neuronal) cells, 10 μM of cytosine arabinoside was added to culture medium at day 1 and kept for 5 consecutive days. The medium was exchanged every day, unless otherwise specified.

### 5.8. Quantitation of CGRP

After 7–10 DIV, the medium was gently aspirated from the TGNs, and 0.25 mL of HEPES buffered saline (HBS, mM: 22.5 HEPES, 135 NaCl, 3.5 KCl, 1 MgCl_2_, 2.5 CaCl_2_, 3.3 glucose, and 0.1% bovine serum albumin (BSA), pH 7.4) was added to each well, and equilibrated at 37 °C for 30 min. For stimulation with capsaicin, a 1 mM stock was prepared in ethanol and diluted in HBS to the required concentration; 0.1% (*v*/*v*) ethanol in HBS served as a vehicle during incubation with HBS for the estimation of non-stimulated exocytosis. To measure the total intracellular content of CGRP, at the end of each experiment, the cells were dissolved into 1% Triton X-100 in HBS on ice for 10–15 min, triturated through a 1 mL pipette tip, centrifuged for 1 min (20,000× *g*, 4 °C) to remove non-solubilized matter and stored at −20 °C until assayed.

To determine the amounts of CGRP released from the TGNs under resting conditions, upon stimulation with capsaicin and in soluble cell lysates, 0.1 mL of each sample was added to 96-well plates coated with a monoclonal antibody specific for CGRP. It was quantified by ELISA, according to the manufacturer’s instructions. Each time an ELISA was performed, a standard curve was generated by serial dilution and assay of a standard sample of CGRP provided with the kit. The results were plotted in GraphPad Prism version 9.2 (San Diego, CA, USA), fit by a linear function, and the equation was then used in Excel (Microsoft Office 2016, St. Redmond, WA, USA) to calculate the CGRP concentrations in test samples. Resting release values obtained for each well were subtracted from those for capsaicin stimulation to yield the evoked component. To facilitate comparisons between experiments, released CGRP was normalized as a % of the total CGRP content (i.e., the sum of released CGRP and the amount in solubilized cell lysates). In some experiments, TGNs were pre-incubated with 100 nM BoNT/A, BoNT/EA, or LC/E-BoNT/A for 48 h at 37 °C (5% CO_2_/95% O_2_) added directly to the culture medium.

### 5.9. Western Blotting and Quantification of SNAP-25 Cleavage

Following completion of the release experiments, 1–2 wells of cells treated with BoNT/A, BoNT/EA, or LC/E-BoNT/A, as well as neurotoxin-free controls, were washed thrice with HBS before being dissolved in LDS sample-buffer. The solutions were then heated at 95 °C for 5 min before electrophoresis on 12% polyacrylamide Bis-Tris Bolt SDS gels. Proteins were transferred onto PVDF membrane using a semi-dry Pierce^TM^ Power Blotter (Thermo Fisher, Cheshire, UK). After blocking with 3% BSA in 50 mM Tris/150 mM NaCl/0.1% Tween^®^ 20, pH 7.6 (TBS-T), the membranes were incubated overnight at 4 °C with a mouse monoclonal antibody (1:3000 in TBS-T) reactive with SNAP-25 and BoNT/A- and /E-truncated forms. After three 10 min washes with TBS, this was followed by exposure to an anti-mouse IgG secondary antibody conjugated to AP (1:10,000) for 1 h at room temperature. The membranes were washed another 3 times with TBS before development of colored product by incubation with a buffered solution containing AP substrates (100 mM Tris, 100 mM NaCl, 5 mM MgCl_2_, 0.165 mg/mL 5-bromo-4-chloro-3-indolyl phosphate, and 0.33 mg/mL nitro blue tetrazolium). Images of the bands that developed were captured using a digital camera and a densitometric analysis was performed using ImageJ software; the resultant data were normalized as indicated in figure legends.

### 5.10. Data Analysis and Statistics

All data were analyzed using GraphPad Prism version 9.2 and presented as mean + standard error of the mean (SEM). Statistical significance among groups was defined as *p* < 0.05 and, where possible, Student’s *t*-test, one-way, or two-way analysis of variance (ANOVA) were applied. Bonferroni’s post hoc test was used to assess comparisons between-groups at individual time points, as appropriate.

## Data Availability

Data sharing not applicable.

## References

[1] Dolly J.O., Meng J., Wang J., Lawrence G.W., Bodeker M., Zurawski T.H., Sasse A., Atassi M.Z. (2009). Multiple Steps in the Blockade of Exocytosis by Botulinum Neurotoxins. Botulinum Toxin: Therapeutic Clinical Practice and Science.

[2] Dolly J.O., Wang J., Zurawski T.H., Meng J. (2011). Novel therapeutics based on recombinant botulinum neurotoxins to normalize the release of transmitters and pain mediators. FEBS J..

[3] Sudhof T.C. (2014). The molecular machinery of neurotransmitter release (Nobel lecture). Angew. Chem. Int. Ed. Engl..

[4] Dong M., Yeh F., Tepp W.H., Dean C., Johnson E.A., Janz R., Chapman E.R. (2006). SV2 is the protein receptor for botulinum neurotoxin A. Science.

[5] Dong M., Liu H., Tepp W.H., Johnson E.A., Janz R., Chapman E.R. (2008). Glycosylated SV2A and SV2B mediate the entry of botulinum neurotoxin E into neurons. Mol. Biol. Cell.

[6] Mahrhold S., Rummel A., Bigalke H., Davletov B., Binz T. (2006). The synaptic vesicle protein 2C mediates the uptake of botulinum neurotoxin A into phrenic nerves. FEBS Lett..

[7] Dolly J.O., Black J., Williams R.S., Melling J. (1984). Acceptors for botulinum neurotoxin reside on motor nerve terminals and mediate its internalization. Nature.

[8] Black J.D., Dolly J.O. (1986). Interaction of 125I-labeled botulinum neurotoxins with nerve terminals. I. Ultrastructural autoradiographic localization and quantitation of distinct membrane acceptors for types A and B on motor nerves. J. Cell Biol..

[9] Black J.D., Dolly J.O. (1986). Interaction of 125I-labeled botulinum neurotoxins with nerve terminals. II. Autoradiographic evidence for its uptake into motor nerves by acceptor-mediated endocytosis. J. Cell Biol..

[10] Rossetto O., Pirazzini M., Fabris F., Montecucco C. (2021). Botulinum Neurotoxins: Mechanism of Action. Handb. Exp. Pharmacol..

[11] Ashton A.C., Dolly J.O. (1988). Characterization of the inhibitory action of botulinum neurotoxin type A on the release of several transmitters from rat cerebrocortical synaptosomes. J. Neurochem..

[12] McMahon H.T., Foran P., Dolly J.O., Verhage M., Wiegant V.M., Nicholls D.G. (1992). Tetanus toxin and botulinum toxins type A and B inhibit glutamate, gamma-aminobutyric acid, aspartate, and met-enkephalin release from synaptosomes. Clues to the locus of action. J. Biol. Chem..

[13] Purkiss J., Welch M., Doward S., Foster K. (2000). Capsaicin-stimulated release of substance P from cultured dorsal root ganglion neurons: Involvement of two distinct mechanisms. Biochem. Pharmacol..

[14] Welch M.J., Purkiss J.R., Foster K.A. (2000). Sensitivity of embryonic rat dorsal root ganglia neurons to Clostridium botulinum neurotoxins. Toxicon.

[15] Durham P.L., Cady R., Cady R. (2004). Regulation of calcitonin gene-related peptide secretion from trigeminal nerve cells by botulinum toxin type A: Implications for migraine therapy. Headache.

[16] Meng J., Wang J., Lawrence G., Dolly J.O. (2007). Synaptobrevin I mediates exocytosis of CGRP from sensory neurons and inhibition by botulinum toxins reflects their anti-nociceptive potential. J. Cell Sci..

[17] Meng J., Ovsepian S.V., Wang J., Pickering M., Sasse A., Aoki K.R., Lawrence G.W., Dolly J.O. (2009). Activation of TRPV1 mediates calcitonin gene-related peptide release, which excites trigeminal sensory neurons and is attenuated by a retargeted botulinum toxin with anti-nociceptive potential. J. Neurosci..

[18] Avona A., Mason B.N., Lackovic J., Wajahat N., Motina M., Quigley L., Burgos-Vega C., Moldovan Loomis C., Garcia-Martinez L.F., Akopian A.N. (2020). Repetitive stress in mice causes migraine-like behaviors and calcitonin gene-related peptide-dependent hyperalgesic priming to a migraine trigger. Pain.

[19] Cui M., Khanijou S., Rubino J., Aoki K.R. (2004). Subcutaneous administration of botulinum toxin A reduces formalin-induced pain. Pain.

[20] Bach-Rojecky L., Relja M., Lackovic Z. (2005). Botulinum toxin type A in experimental neuropathic pain. J. Neural Transm. (Vienna).

[21] Park H.J., Lee Y., Lee J., Park C., Moon D.E. (2006). The effects of botulinum toxin A on mechanical and cold allodynia in a rat model of neuropathic pain. Can. J. Anaesth..

[22] Zychowska M., Rojewska E., Makuch W., Luvisetto S., Pavone F., Marinelli S., Przewlocka B., Mika J. (2016). Participation of pro- and anti-nociceptive interleukins in botulinum toxin A-induced analgesia in a rat model of neuropathic pain. Eur. J. Pharmacol..

[23] Xiao L., Cheng J., Zhuang Y., Qu W., Muir J., Liang H., Zhang D. (2013). Botulinum toxin type A reduces hyperalgesia and TRPV1 expression in rats with neuropathic pain. Pain Med..

[24] Filipovic B., Matak I., Bach-Rojecky L., Lackovic Z. (2012). Central action of peripherally applied botulinum toxin type A on pain and dural protein extravasation in rat model of trigeminal neuropathy. PLoS ONE.

[25] Shimizu T., Shibata M., Toriumi H., Iwashita T., Funakubo M., Sato H., Kuroi T., Ebine T., Koizumi K., Suzuki N. (2012). Reduction of TRPV1 expression in the trigeminal system by botulinum neurotoxin type-A. Neurobiol. Dis..

[26] Caterina M.J., Schumacher M.A., Tominaga M., Rosen T.A., Levine J.D., Julius D. (1997). The capsaicin receptor: A heat-activated ion channel in the pain pathway. Nature.

[27] Basith S., Cui M., Hong S., Choi S. (2016). Harnessing the Therapeutic Potential of Capsaicin and Its Analogues in Pain and Other Diseases. Molecules.

[28] Burstein R., Blumenfeld A.M., Silberstein S.D., Manack Adams A., Brin M.F. (2020). Mechanism of Action of OnabotulinumtoxinA in Chronic Migraine: A Narrative Review. Headache.

[29] Aurora S.K., Dodick D.W., Turkel C.C., DeGryse R.E., Silberstein S.D., Lipton R.B., Diener H.C., Brin M.F., Group P.C.M.S. (2010). OnabotulinumtoxinA for treatment of chronic migraine: Results from the double-blind, randomized, placebo-controlled phase of the PREEMPT 1 trial. Cephalalgia.

[30] Diener H.C., Dodick D.W., Aurora S.K., Turkel C.C., DeGryse R.E., Lipton R.B., Silberstein S.D., Brin M.F., Group P.C.M.S. (2010). OnabotulinumtoxinA for treatment of chronic migraine: Results from the double-blind, randomized, placebo-controlled phase of the PREEMPT 2 trial. Cephalalgia.

[31] Dodick D.W., Turkel C.C., DeGryse R.E., Aurora S.K., Silberstein S.D., Lipton R.B., Diener H.C., Brin M.F., Group P.C.M.S. (2010). OnabotulinumtoxinA for treatment of chronic migraine: Pooled results from the double-blind, randomized, placebo-controlled phases of the PREEMPT clinical program. Headache.

[32] Khalil M., Zafar H.W., Quarshie V., Ahmed F. (2014). Prospective analysis of the use of OnabotulinumtoxinA (BOTOX) in the treatment of chronic migraine; real-life data in 254 patients from Hull, U.K. J. Headache Pain.

[33] Dominguez C., Pozo-Rosich P., Torres-Ferrus M., Hernandez-Beltran N., Jurado-Cobo C., Gonzalez-Oria C., Santos S., Monzon M.J., Latorre G., Alvaro L.C. (2018). OnabotulinumtoxinA in chronic migraine: Predictors of response. A prospective multicentre descriptive study. Eur. J. Neurol..

[34] Cernuda-Morollon E., Ramon C., Martinez-Camblor P., Serrano-Pertierra E., Larrosa D., Pascual J. (2015). OnabotulinumtoxinA decreases interictal CGRP plasma levels in patients with chronic migraine. Pain.

[35] Lassen L.H., Jacobsen V.B., Haderslev P.A., Sperling B., Iversen H.K., Olesen J., Tfelt-Hansen P. (2008). Involvement of calcitonin gene-related peptide in migraine: Regional cerebral blood flow and blood flow velocity in migraine patients. J. Headache Pain.

[36] Hansen J.M., Hauge A.W., Olesen J., Ashina M. (2010). Calcitonin gene-related peptide triggers migraine-like attacks in patients with migraine with aura. Cephalalgia.

[37] Benemei S., Dussor G. (2019). TRP Channels and Migraine: Recent Developments and New Therapeutic Opportunities. Pharmaceuticals (Basel).

[38] Maraia Z., Ricci D., Rocchi M.B.L., Moretti A., Bufarini C., Cavaliere A., Peverini M. (2021). Real-Life Analysis with Erenumab: First Target Therapy in the Episodic and Chronic Migraine’s Prophylaxis. J. Clin. Med..

[39] Edvinsson L., Haanes K.A., Warfvinge K., Krause D.N. (2018). CGRP as the target of new migraine therapies - successful translation from bench to clinic. Nat. Rev. Neurol..

[40] Goadsby P.J., Holland P.R., Martins-Oliveira M., Hoffmann J., Schankin C., Akerman S. (2017). Pathophysiology of Migraine: A Disorder of Sensory Processing. Physiol. Rev..

[41] Wang J., Meng J., Lawrence G.W., Zurawski T.H., Sasse A., Bodeker M.O., Gilmore M.A., Fernandez-Salas E., Francis J., Steward L.E. (2008). Novel chimeras of botulinum neurotoxins A and E unveil contributions from the binding, translocation, and protease domains to their functional characteristics. J. Biol. Chem..

[42] Wang J., Casals-Diaz L., Zurawski T., Meng J., Moriarty O., Nealon J., Edupuganti O.P., Dolly O. (2017). A novel therapeutic with two SNAP-25 inactivating proteases shows long-lasting anti-hyperalgesic activity in a rat model of neuropathic pain. Neuropharmacology.

[43] Gambeta E., Chichorro J.G., Zamponi G.W. (2020). Trigeminal neuralgia: An overview from pathophysiology to pharmacological treatments. Mol. Pain.

[44] Ossipov M.H., Dussor G.O., Porreca F. (2010). Central modulation of pain. J. Clin. Investig..

[45] Chichorro J.G., Porreca F., Sessle B. (2017). Mechanisms of craniofacial pain. Cephalalgia.

[46] Deuis J.R., Dvorakova L.S., Vetter I. (2017). Methods Used to Evaluate Pain Behaviors in Rodents. Front. Mol. Neurosci..

[47] Munro G., Jansen-Olesen I., Olesen J. (2017). Animal models of pain and migraine in drug discovery. Drug Discov. Today.

[48] Percie du Sert N., Rice A.S. (2014). Improving the translation of analgesic drugs to the clinic: Animal models of neuropathic pain. Br. J. Pharmacol..

[49] Heinz D.E., Schottle V.A., Nemcova P., Binder F.P., Ebert T., Domschke K., Wotjak C.T. (2021). Exploratory drive, fear, and anxiety are dissociable and independent components in foraging mice. Transl. Psychiatry.

[50] Coe M.A., Lofwall M.R., Walsh S.L. (2019). Buprenorphine Pharmacology Review: Update on Transmucosal and Long-acting Formulations. J. Addict. Med..

[51] Vuralli D., Wattiez A.S., Russo A.F., Bolay H. (2019). Behavioral and cognitive animal models in headache research. J. Headache Pain.

[52] Miyashita S.I., Zhang J., Zhang S., Shoemaker C.B., Dong M. (2021). Delivery of single-domain antibodies into neurons using a chimeric toxin-based platform is therapeutic in mouse models of botulism. Sci. Transl. Med..

[53] Bach-Rojecky L., Lackovic Z. (2005). Antinociceptive effect of botulinum toxin type a in rat model of carrageenan and capsaicin induced pain. Croat. Med. J..

[54] Luvisetto S., Vacca V., Cianchetti C. (2015). Analgesic effects of botulinum neurotoxin type A in a model of allyl isothiocyanate- and capsaicin-induced pain in mice. Toxicon.

[55] Mogil J.S. (2020). Qualitative sex differences in pain processing: Emerging evidence of a biased literature. Nat. Rev. Neurosci..

[56] Doyle H.H., Eidson L.N., Sinkiewicz D.M., Murphy A.Z. (2017). Sex Differences in Microglia Activity within the Periaqueductal Gray of the Rat: A Potential Mechanism Driving the Dimorphic Effects of Morphine. J. Neurosci..

[57] Inyang K.E., Szabo-Pardi T., Wentworth E., McDougal T.A., Dussor G., Burton M.D., Price T.J. (2019). The antidiabetic drug metformin prevents and reverses neuropathic pain and spinal cord microglial activation in male but not female mice. Pharmacol. Res..

[58] Hunt S.P., Pini A., Evan G. (1987). Induction of c-fos-like protein in spinal cord neurons following sensory stimulation. Nature.

[59] Harriott A.M., Strother L.C., Vila-Pueyo M., Holland P.R. (2019). Animal models of migraine and experimental techniques used to examine trigeminal sensory processing. J. Headache Pain.

[60] Hegarty D.M., Hermes S.M., Largent-Milnes T.M., Aicher S.A. (2014). Capsaicin-responsive corneal afferents do not contain TRPV1 at their central terminals in trigeminal nucleus caudalis in rats. J. Chem. Neuroanat..

[61] Mangione A.S., Obara I., Maiaru M., Geranton S.M., Tassorelli C., Ferrari E., Leese C., Davletov B., Hunt S.P. (2016). Nonparalytic botulinum molecules for the control of pain. Pain.

[62] Matak I., Bach-Rojecky L., Filipovic B., Lackovic Z. (2011). Behavioral and immunohistochemical evidence for central antinociceptive activity of botulinum toxin A. Neuroscience.

[63] Matak I., Rossetto O., Lackovic Z. (2014). Botulinum toxin type A selectivity for certain types of pain is associated with capsaicin-sensitive neurons. Pain.

[64] Lovrencic L., Matak I., Lackovic Z. (2020). Association of Intranasal and Neurogenic Dural Inflammation in Experimental Acute Rhinosinusitis. Front. Pharmacol..

[65] Cha M., Sallem I., Jang H.W., Jung I.Y. (2020). Role of transient receptor potential vanilloid type 1 in the trigeminal ganglion and brain stem following dental pulp inflammation. Int. Endod. J..

[66] Matak I., Bolcskei K., Bach-Rojecky L., Helyes Z. (2019). Mechanisms of Botulinum Toxin Type A Action on Pain. Toxins (Basel).

[67] Ramachandran R., Yaksh T.L. (2014). Therapeutic use of botulinum toxin in migraine: Mechanisms of action. Br. J. Pharmacol..

[68] Julius D. (2013). TRP channels and pain. Annu. Rev. Cell Dev. Biol..

[69] Cholewinski A., Burgess G.M., Bevan S. (1993). The role of calcium in capsaicin-induced desensitization in rat cultured dorsal root ganglion neurons. Neuroscience.

[70] Kim Y.S., Chu Y., Han L., Li M., Li Z., LaVinka P.C., Sun S., Tang Z., Park K., Caterina M.J. (2014). Central terminal sensitization of TRPV1 by descending serotonergic facilitation modulates chronic pain. Neuron.

[71] Lawrence G.W., Zurawski T.H., Dong X., Dolly J.O. (2021). Population Coding of Capsaicin Concentration by Sensory Neurons Revealed Using Ca(2+) Imaging of Dorsal Root Ganglia Explants from Adult pirt-GCaMP3 Mouse. Cell. Physiol. Biochem..

[72] Baker P.F., Knight D.E. (1978). Calcium-dependent exocytosis in bovine adrenal medullary cells with leaky plasma membranes. Nature.

[73] Meng J., Dolly J.O., Wang J. (2014). Selective cleavage of SNAREs in sensory neurons unveils protein complexes mediating peptide exocytosis triggered by different stimuli. Mol. Neurobiol..

[74] Molgo J., Thesleff S. (1984). Studies on the mode of action of botulinum toxin type A at the frog neuromuscular junction. Brain Res..

[75] Hayashi T., McMahon H., Yamasaki S., Binz T., Hata Y., Sudhof T.C., Niemann H. (1994). Synaptic vesicle membrane fusion complex: Action of clostridial neurotoxins on assembly. EMBO J..

[76] Otto H., Hanson P.I., Chapman E.R., Blasi J., Jahn R. (1995). Poisoning by botulinum neurotoxin A does not inhibit formation or disassembly of the synaptosomal fusion complex. Biochem. Biophys. Res. Commun..

[77] Wang J., Zurawski T.H., Meng J., Lawrence G., Olango W.M., Finn D.P., Wheeler L., Dolly J.O. (2011). A dileucine in the protease of botulinum toxin A underlies its long-lived neuroparalysis: Transfer of longevity to a novel potential therapeutic. J. Biol. Chem..

[78] Khounlo R., Kim J., Yin L., Shin Y.K. (2017). Botulinum Toxins A and E Inflict Dynamic Destabilization on t-SNARE to Impair SNARE Assembly and Membrane Fusion. Structure.

[79] Sakaba T., Stein A., Jahn R., Neher E. (2005). Distinct kinetic changes in neurotransmitter release after SNARE protein cleavage. Science.

[80] Percie du Sert N., Ahluwalia A., Alam S., Avey M.T., Baker M., Browne W.J., Clark A., Cuthill I.C., Dirnagl U., Emerson M. (2020). Reporting animal research: Explanation and elaboration for the ARRIVE guidelines 2.0. PLoS Biol..

[81] Wang J., Meng J., Nugent M., Tang M., Dolly J.O. (2017). Neuronal entry and high neurotoxicity of botulinum neurotoxin A require its N-terminal binding sub-domain. Sci. Rep..

[82] Romero-Reyes M., Akerman S., Nguyen E., Vijjeswarapu A., Hom B., Dong H.W., Charles A.C. (2013). Spontaneous behavioral responses in the orofacial region: A model of trigeminal pain in mouse. Headache.

[83] Paxinos G., Watson C. (2005). The Rat Brain in Stereotaxic Coordinates.

[84] Eckert S.P., Taddese A., McCleskey E.W. (1997). Isolation and culture of rat sensory neurons having distinct sensory modalities. J. Neurosci. Methods.

